# The Association Between Rheumatic Disease Therapies and Cardiovascular Outcomes in People with HIV—A Retrospective Cohort Study

**DOI:** 10.3390/jcm13206209

**Published:** 2024-10-18

**Authors:** Boghuma K. Titanji, Shumpei Nagatomi, Julia W. Gallini, Xiangqin Cui, Jennifer S. Hanberg, Evelyn Hsieh, Vincent C. Marconi

**Affiliations:** 1 Division of Infectious Diseases, Emory University, Atlanta, GA 30322, USA; btitanj@emory.edu; 2 Department of Epidemiology and Biostatistics, Rollins School of Public Health, Emory University, Atlanta, GA 30322, USA; shumpei-nagatomi@saitama-sekishinkai.org (S.N.); xiangqin.cui@emory.edu (X.C.); 3 Veterans Affairs Medical Center, Decatur, GA 30033, USA; 4 Department of Medicine, Massachusetts General Hospital, Boston, MA 02114, USA; jhanberg@bwh.harvard.edu; 5 Section of Rheumatology, Allergy & Immunology, Department of Internal Medicine, Yale University School of Medicine, New Haven, CT 06510, USA; 6 Veterans Affairs Connecticut Healthcare System, West Haven VA Medical Center, West Haven, CT 06516, USA; 7 Hubert Department of Global Health, Rollins School of Public Health, Emory University, Atlanta, GA 30329, USA; 8 Emory Vaccine Center, Yerkes National Primate Research Center, Emory University, Atlanta, GA 30317, USA

**Keywords:** rheumatic disease, HIV, cardiovascular events

## Abstract

**Introduction:** Inflammation is a significant contributor to cardiovascular disease (CVD) in people with HIV (PWH), who face twice the risk of CVD compared to the general population. The presence of co-existing rheumatic disease (RD) may further exacerbate inflammation and increase the incidence of CVD events in this population. **Methods**: We conducted a retrospective cohort study using electronic health record (EHR) data from the Veterans Affairs Medical Center in Atlanta, covering the period from 2000 to 2019. A total of 5000 patients aged 20–87 years who were diagnosed with HIV and receiving care at the Atlanta VAMC between 2000 and 2019 were eligible for this analysis. This study included 3930 veterans with HIV and assessed the impact of rheumatic disease therapies (RDTs) on CVD outcomes. The primary outcome was the first occurrence of a CVD event. **Results:** Rheumatic disease was significantly associated with an increased risk of CVD events (OR = 2.67; *p* < 0.001). Additionally, exposure to multiple RDTs (aHR = 2.121, *p* = 0.047), NSAIDs (aHR = 1.694, *p* = 0.003), glucocorticoids (aHR = 2.332, *p* < 0.0001), and hypouricemic agents and colchicine (aHR = 3.445, *p* < 0.0001) were all significantly associated with increased CVD events. **Conclusions:** The co-existence of HIV infection and rheumatic disease, along with the use of RDTs, may amplify the risk of CVD events in PWH. These findings underscore the need for further investigation into the relationship between RD, RDTs, and CVD risk in larger, controlled studies, given the potential implications for treatment decisions in this patient population. A limitation of our study is that due to its retrospective design, we could not examine the impact of the sequential use of RDT groups and RD severity on CVD events.

## 1. Introduction

Antiretroviral therapy (ART) has improved clinical outcomes for people with HIV (PWH). The chronic inflammation and immunologic dysregulation associated with HIV infection and the prolonged lifespan as a result of ART use predispose patients in this population to a higher burden of chronic diseases and comorbidities [1]. Several clinical studies have assessed targeting inflammation as a way of mitigating the overall cardiovascular disease (CVD) risk profile and comorbidities for PWH [2], with limited success. Through related mechanisms, rheumatic diseases (RDs) are also independently associated with an increased risk of CVD [3], which is not fully explained by traditional risk factors. Chronic inflammation associated with HIV and with rheumatic diseases contributes to endothelial dysfunction, which is an important precursor for atherogenesis and atherosclerotic cardiovascular disease. Co-existent RD and HIV have the potential to exacerbate chronic inflammation, with risks of poor cardiovascular outcomes. Rheumatologic disease therapies (RDTs) are directed at reducing inflammation and targeting specific immunologic pathways that drive disease. These, therefore, have the potential to indirectly impact atherogenesis and cardiovascular outcomes through their effects on inflammation. The aims of this study were to explore the association of RD and RDT on CVD events among PWH in a retrospective cohort of U.S. Veterans.

## 2. Methods

This was a retrospective cohort study based on electronic health record (EHR) data collected from the Veterans Affairs Medical Center (VAMC) Corporate Data Warehouse (CDW). A total of 5000 patients aged 20–87 years who were diagnosed with HIV and receiving care at the Atlanta VAMC between 2000 and 2019 were eligible for this analysis. The Emory University institutional review board and VAMC Research and Development Committee approved this study (IRB IDNo:MOD0027-IRB00068877) and issued a consent waiver. Fully anonymized patient data were abstracted between January 2021 and December 2022. Individuals who had experienced a cardiovascular event before their HIV diagnosis, those who experienced a cardiovascular event during the two-year exposure period, and those who died prior to the year 2000 or during the exposure period were excluded (Figures S1 and S3). The international classification for disease (ICD)9/10 codes for RD diagnoses were used to identify eligible participants with RD (Table S2). Twenty randomly selected charts (selected from the 362 charts identified as participants with RD) were reviewed to confirm that the ICD codes adequately captured participants with RD, and a concordance of 95% was achieved (19/20 charts). Baseline covariates included race, ethnicity, hypertension, diabetes mellitus, smoking, low-dose aspirin, ART and the Veterans Aging Cohort Study Index (VACS index), and a validated disease burden index for PWH (incorporating CD4+ T cell count, HIV viral load, Platelet count, HCV status, and age) [4]. The exposure variable, use of RDT at any time (Table S3), was categorized into six groups: (1) non-steroidal anti-inflammatory drugs (NSAIDs), (2) glucocorticoids, (3) disease-modifying anti-rheumatic drugs (DMARDs), (4) hypouricemic agents and colchicine, (5) multiple RDTs (participants who received concurrent or sequential medications from multiple classes), and (6) no RDT. The primary outcome was the first occurrence of a CVD event occurring at least two years after HIV diagnosis, identified by the ICD9/10 codes and categorized as (1) myocardial infarction (MI), (2) stroke, (3) congestive heart failure (CHF), and (4) peripheral arterial disease (PAD) (Table S4).

### Statistical Analyses

A chi-squared test was applied to determine the association between CVD events and RD, and the odds ratio (OR) was calculated. Competing risks modeling (Fine and Gray) was used to identify multivariate predictors of time to CVD events among the proposed covariates, with the first occurrence of a CVD event being used as the endpoint and the RDT category as the main exposure. Event-free survival was calculated two years after the date of HIV diagnosis to the date of the most recent CD4+ T cell count result (last date recorded in the EHR), the date of CVD diagnosis, or the date of death from any cause. We also stratified the participants by RD status. The cumulative incidence curves were compared by exposure group for CVD events using Gray’s test [5] (Figure S2). For all tests described, a *p*-value <0.05 was considered statistically significant. All analyses were performed using SAS version 9.4.

## 3. Results

### Baseline Characteristics

Of the 5000 eligible screened patients, 3930 participants were included in our analyses (Figure S3). The median follow-up time was 7.59 (SD: 3.26–13.52) years. The median age of the cohort was 44 (IQR: 36–52) years. The study population was 96.6% male, 70% African American/Black, and 20% Caucasian. The median VACS index score was 23 (IQR: 12–40). Descriptive statistics are summarized in Table 1.

Of the participants included in the study, 362 (9.2%) had an RD diagnosis. Of note, 21.7% of the participants had a smoking history, 19.9% had a diagnosis of hypertension, and 9% had a diagnosis of hyperlipidemia. A total of 660 first CVD events were observed in the cohort: 264 MI (40%), 110 stroke (16.7%), 180 CHF (27.3%), and 160 PAD (16.1%). There was a statistically significant association between RD and having a first CVD event (OR = 2.67; *p* < 0.001). In the overall group, based on Gray’s test, there were significant differences between the RDT groups (*p* < 0.001) and risk for a first CVD event (Figure S2). A multivariate Fine and Gray model was used to identify independent predictors of time to CVD events. Compared to the no-RDT group, participants exposed to multiple RDTs (HR = 2.121, 95% CI 1.26–3.6), NSAIDs (HR = 1.694, 95% CI 1.2–2.4), glucocorticoids (HR = 2.332, 95% CI 1.58–3.45), or hypouricemic agents (HR = 3.445, 95% CI 2.14–5.55) were significantly more likely to experience a CVD event after adjusting for RD status and key covariates (gender, race, ART, VACS index, RD status, hypertension, hyperlipidemia, smoking, and baby aspirin use). The effect of the DMARD group was not statistically significant (Table 2).

After stratifying by the presence of RD, the significant association between RDT and CVD events persisted only in PWH without RD (i.e., people with HIV who received RDT for indications besides rheumatologic disease): multiple RDTs (HR = 1.97, 95% CI 1.03–3.77), NSAIDs (HR = 1.75, 95% CI 1.22–2.51), glucocorticoids (HR = 2.46, 95% CI 1.64–3.7), hypouricemic agents, and colchicine (HR = 2.96) (Table S1).

## 4. Discussion

In this retrospective study, the impact of RDT on incident CVD events among PWH was examined for the first time. Our results showed that the use of multiple RDTs, NSAIDs, glucocorticoids, and hypouricemic agents and colchicine were associated with an increased hazard for CVD events compared to no RD medications. Glucocorticoids are frequently prescribed but have a wide range of metabolic effects that are relevant for CVD. In one study using a general practice research database of individuals without HIV, Souverein et al. matched 50,656 cases to an equivalent number of age-matched controls and reported that oral glucocorticoids increased the odds of CHF (OR 2.66; CI 2.46–2.87) and, to a lesser degree, atherosclerosis and cerebrovascular disease [6]. Non-selective and cyclooxygenase-2 NSAIDs also increase the risk of CVD events in the general population [7,8,9]. Their use has been associated with cardiovascular death, MI, and stroke. Glucocorticoids and NSAIDs are frequently prescribed for a variety of indications besides rheumatologic conditions for PWH, but no previous studies have examined how these therapies impact the overall CVD risk and CVD events in this population. Our results suggest that glucocorticoid and NSAIDs were significantly associated with CVD events in PWH, and this may need to be considered when prescribing these medications for this population.

Hypouricemic agents and colchicine have garnered interest as potentially useful strategies for reducing CVD risk [10,11,12,13,14,15,16]. Allopurinol, in a small randomized controlled trial (RCT) reduced nitric oxide levels and endothelial damage [11]. Colchicine—an anti-inflammatory drug frequently combined with hypouricemic agents in the treatment of gout—has been shown in multiple RCTs to reduce the risk of CVD events in patients with coronary artery disease [12,13,16]. In our study, hypouricemic agents and colchicine were considered together as a medication group and found to be associated with an increased risk of CVD events. Due to small numbers in this RDT group, we were limited in our ability to evaluate each medication separately. One possible explanation is confounding by indication, since individuals with hyperuricemia are more likely to have CVD events. Another possibility is that the mechanisms by which HIV mediates CVD risk may not be impacted by uric acid-lowering drugs or colchicine in the same way as in HIV-negative cohorts. Only 17 individuals received treatment with a synthetic or biologic DMARD, and among these, 6 had a diagnosis of RD, suggesting that some patients may have been treated for other related conditions. Although the aHR for CVD events was increased, this association was not statistically significant. The infrequent use of DMARD in this cohort of PWH may reflect prescription practices which tend to avoid the use of these medications in people who have underlying immunocompromised states, given limited experience and increased risk of interactions with ART [17,18]. As a result, we speculate that suboptimal control of concurrent RD may contribute to increased risk of CVD events in PWH. Additionally, we did not evaluate medication adherence to ART or RDTs in our study, another important factor which could impact on the outcomes observed. It is well established that as people with HIV age, they are disproportionately impacted by chronic co-morbid conditions that are frequently associated with polypharmacy. It is important to encourage clinicians to consider the effects that RDTs could have on potentially worsening CVD outcomes, and this requires better characterization in future studies, including relevant control populations.

## 5. Limitations

Firstly, the lack of an HIV-negative comparator group limits our ability to interpret these findings in the context of the general population. Secondly, the cohort is predominantly male, and consequently, the results cannot be extrapolated to women with HIV. Thirdly, the sample size limited our ability to fully evaluate the impact of DMARDs on CVD outcomes. Fourthly, we used data mined from the EHR, which may be limited by the heterogeneity and the accuracy of recorded diagnoses. Additionally, due to the study design, we could not examine the impact of sequential use of RDT groups and RD severity on CVD events. Lastly, we recognize that the term ‘rheumatic diseases’ encompasses a broad spectrum of conditions, each with distinct pathophysiological mechanisms that may influence inflammation and cardiovascular risk in unique ways. Future studies with larger sample sizes should aim to investigate specific rheumatic diseases individually to more accurately assess how treatment strategies for these conditions impact cardiovascular outcomes in PWH.

## 6. Conclusions

Our study found that co-morbid RD in PWH may by associated with increased CVD events and provides initial data examining the relationship between RDT and CVD risk for PWH with and without RD. These findings highlight the need to further explore the relationship between RD, RDTs, and CVD risk in larger, controlled studies given the possible implications for treatment choices in this patient population. Understanding how these treatments impact outcomes among people with HIV is especially critical, as their effects have not been previously assessed in studies focusing on RDTs and CVD outcomes. Continued investigation into these associations will be essential for optimizing clinical care and improving health outcomes for individuals living with HIV.

## Figures and Tables

**Table 1:** Baseline characteristics.

Variable	Sub-Category	Participants N (%)
		N = 3930
Median Age (IQR)—yr		44 (36–52)
Gender	Male	3800 (96.6)
	Female	130 (3.3)
Race	Asian	3 (0.08)
	African American	2877 (73.2)
	Native Hawaiian	12 (0.31)
	Unknown	229 (5.8)
	White	796 (20.2)
	American Indian or Alaska	13 (0.33)
Antiretroviral drug	NRTI	926 (28.1)
	NNRTI	180 (5.4)
	PI	494 (15.0)
	INSTI	238 (7.2)
	Gp41 antagonist	1 (0.03)
	CCR5 antagonist	5 (0.15)
	NRTI/NNRTI	317 (9.6)
	NRTI/PI	21 (0.64)
	NRTI/INSTI	1078 (32.7)
	NNRTI/INSTI	34 (1.03)
Hypertension	Yes	744 (19.9)
	No	2986 (80.0)
Hyperlipidemia	Yes	337 (9.0)
	No	3393 (90.9)
Smoking	Yes	811(21.7)
	No	2919 (78.2)
Low-dose aspirin	Yes	124 (3.3)
	No	3606 (96.6)
Diabetes	Yes	80 (2.1)
	No	3568 (9.8)
RD	Yes	362 (9.2)
	No	3595 (90.8)
VACS score (IQR)		23 (12–40)
	Yes	124 (3.3)

Abbreviations: IQR—Interquartile range, RD—rheumatic disease, Gp—glycoprotein, CCR—cellular chemokine receptor, VACS—Veterans Aging Cohort Study, PI—protease inhibitor, NNRTI—non-nucleoside reverse transcriptase inhibitor, NRTI—nucleoside reverse transcriptase inhibitor.

**Table 2:** Adjusted hazard ratios (aHRs) for CVD outcomes associated with RDT exposure and CVD risk factors from the multivariate Fine and Gray model.

Variable	No.	HR	95% CI		*p*-Value
RDT category (Reference group = no RDT)					
Multiple RDT	210	**2.121**	**1.259**	**3.575**	**0.0047**
NSAIDs	2216	**1.694**	**1.196**	**2.400**	**0.0030**
Glucocorticoids	585	**2.332**	**1.575**	**3.452**	**<0.0001**
DMARD	17	1.539	0.392	6.041	0.5368
Hypouricemic agents and colchicine	105	**3.445**	**2.137**	**5.553**	**<0.0001**
Covariates					
RD	362	1.154	0.823	1.620	0.4048
Smoking	811	2.212	**1.581**	**3.095**	**<0.0001**
Hypertension	744	1.257	0.924	1.712	0.1372
Hyperlipidemia	337	0.779	0.487	1.248	0.2939
Diabetes	80	2.352	0.967	5.747	0.0591
Aspirin	124	**2.127**	**1.212**	**3.731**	**0.0077**
VACS Index	3930	**1.014**	**1.010**	**1.019**	**<0.0001**
Male sex	3800	1.257	0.6337	2.493	0.5120

Abbreviations: RD—rheumatic disease, VACS—Veterans Aging Cohort Study, DMARD—disease-modifying anti-rheumatic medication, NSAIDs—non-steroidal anti-inflammatory drugs, HR—hazard ratio, CI—confidence interval, No.—number.

## Data Availability

All data relevant to the study are included in the article and its appendices. Any requests for additional data are subject to review by the VAMC Research and Development Committee.

## Supplementary Materials

The following supporting information can be downloaded at: https://www.mdpi.com/article/10.3390/jcm13206209/s1, Figure S1: Study design; Figure S2: Cumulative incidence curves for CVD compared by exposure group; Figure S3: Study flow diagram showing breakdown of study participants excluded and those included in the analysis, classified by medication exposure category; Table S1: Adjusted hazard ratios (HRs) for CVD events associated with RDT exposure, stratified by RD status; Table S2: ICD-9 and ICD-10 codes for rheumatic disease (RD) conditions; Table S3: List and codes of rheumatic medication categories used in VA medical system; Table S4: ICD-9 and ICD-10 codes for cardiovascular disease outcomes; Table S5: Rheumatic disease diagnoses of study participants included in the study.
