# Supplementary material for: The Association Between Rheumatic Disease Therapies and Cardiovascular Outcomes in People with HIV—A Retrospective Cohort Study
Source: J Clin Med. 2024 Oct 18;13(20):6209. doi: 10.3390/jcm13206209 (PMC11508247; doi:10.3390/jcm13206209)
Supplement: Supplementary file 1 [file jcm-13-06209-s001.zip › jcm-3217685-supplementary.pdf]

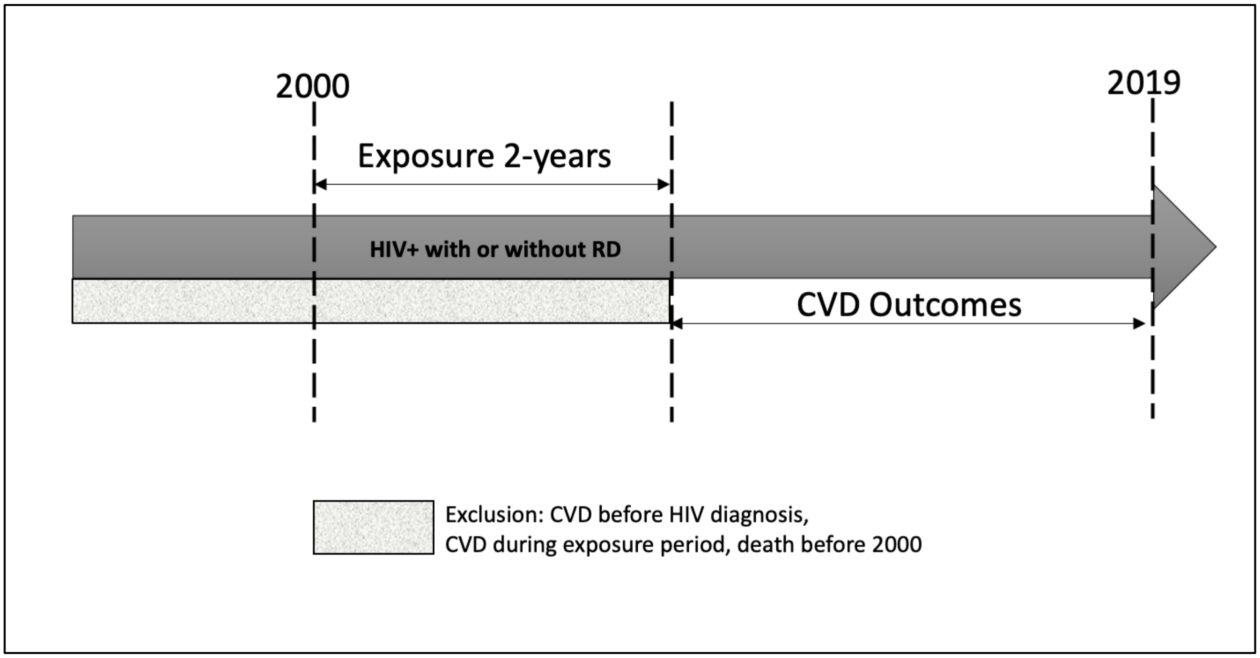

Figure S1. Study design. Legend: Overview of study design—retrospective cohort of US veterans with HIV.

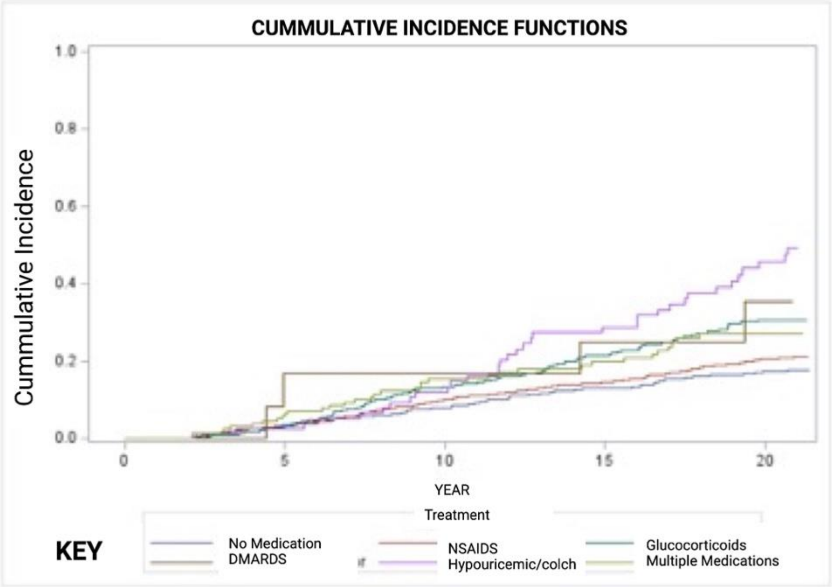

Figure S2. Cumulative incidence curves for CVD compared by exposure group.

Table S1. Adjusted hazard ratios (HRs) for CVD events associated with RDT exposure, stratified by RD status.

Exposure	No.	HR	95%CI		p-value
**Without RD diagnosis**					
Multiple medications	160	**1.969**	**1.029**	**3.766**	**0.0379**
NSAIDs	2072	**1.750**	**1.218**	**2.514**	**0.0030**
Glucocorticoids	529	**2.464**	**1.640**	**3.701**	**<.0001**
DMARDs	11	1.229	0.172	8.766	0.8411
Hypouricemic agents and colchicine	26	2.957	0.951	9.192	0.0626
**With RD diagnosis**					
Multiple medications	50	1.287	0.341	4.852	0.6567
NSAIDs	144	0.936	0.263	3.324	0.9562
Glucocorticoids	56	1.075	0.271	4.266	0.7904
DMARDs	6	1.095	0.130	9.227	0.9427
Hypouricemic agents and colchicine	79	1.779	0.341	4.852	0.3412

Abbreviations: RD—rheumatic disease, VACS—Veterans Aging Cohort Study, DMARD—disease-modifying anti-rheumatic medication, NSAIDs—non-steroidal anti-inflammatory drugs, HR—hazard ratio, CI—confidence interval, No.—number.

Table S2. ICD-9 and ICD-10 codes for rheumatic disease (RD) conditions.

Rheumatic Disease	ICD9	ICD10
Gout, Unspecified	274.9	M10.9
Other disorders of calcium metabolism	275.49	E83.59
Other specified disorders involving the immune mechanism	279.8	D89.89
Psoriatic arthropathy	696.0	L40.54
Other psoriatic arthropathy	696.1	L40.59

SLE	710	M32.10
Progressive systemic sclerosis	710.1	M34.0
Sjögren syndrome	710.2	M35.00
Dermato-polymyositis	710.3	M33.90
Systemic involvement of connective tissue	710.1	M35.9
Crystal arthropathy, unspecified	712.90	M11.9
Rheumatoid arthritis	714.0	M06.9
Unspecified juvenile rheumatoid arthritis	714.3	M08.00
Polymyalgia rheumatica	725	M35.3
Sicca syndrome	710.2	M35.00

Abbreviations: Systemic Lupus erythematosus, ICD—International Classification for Disease.

Table S3. List and codes of rheumatic medication categories used in VA medical system.

**Drug**	**VA class**
Immuno-suppressant	IM600
Immunological agents (other)	IM900
Antirheumatics	MS100
Salicylates, antirheumatic	MS101
Non-salicylates, NSAIDs, anti-rheumatics	MS102
Anti-gout agents	MS400
Glucocorticoids	HS051
Anti-rheumatics, other	MS190
Anti-neoplastic, antimetabolites	AN300
Sulfonamides/related antimicrobials	AM650

Table S4. ICD-9 and ICD-10 codes for cardiovascular disease outcomes.

Diagnosis	ICD-9	ICD-10
Cardiovascular disease, unspecified	429.2	I25.10
Congestive heart failure, unspecified	428	I50.9
Acute, but ill-defined, cerebrovascular disease	436	I63.9
Cerebral artery occlusion, unspecified with cerebral infarction	434.91	I65.29,
Occlusion and stenosis of carotid artery without mention of cerebral infarction	433.1	I65.23, I67.9
Cerebral atherosclerosis	437	I67.2
Peripheral vascular disease, unspecified	443.9	I73.9
Angina pectoris	413	I20.9
Acute myocardial infarction	410	I21.09. I21.3
Old myocardial infarction	412	I25.2
Coronary atherosclerosis	414	I25.10
Chronic ischemic heart disease, unspecified	414.9	I125.9

Abbreviations: ICD—International Classification for Disease.

Table S5. Rheumatic disease diagnoses of study participants included in the study.

Diagnosis	N (%)
Autoimmune hepatitis	4 (1.1)
Biliary Cirrhosis	4 (1.1)
Celiac disease	4 (1.1)
Cholangitis	6 (1.7)
Crohns' disease unspecified, without complications	10 (2.8)
Crystal arthropathy, unspecified	2 (0.6)
Drug-Induced Autoimmune hemolytic anemia	1 (0.3)

Gout arthropathy	225 (62.2)
Psoriatic arthropathy	3 (0.8)
Other Psoriasis	14 (3.8)
Psoriasis vulgaris	8 (2.2)
Systemic lupus erythematosus	2 (0.6)
Regional enteritis	13 (3.5)
Rheumatoid arthritis, unspecified	7 (1.9)
Sicca syndrome	9 (2.5)
Systemic Sclerosis	2 (0.6)
Sarcoidosis, unspecified	9 (2.5)
Systemic involvement of connective tissue, unspecified	1 (0.3)
Ulcerative colitis	17 (4.6)
Other hemolytic anemias	1 (0.3)
Other Disorders of Calcium Metabolism	16 (4.4)
Other unspecified disorders involving the immune mechanism	4 (1.1)
**Total**	**362 (100)**

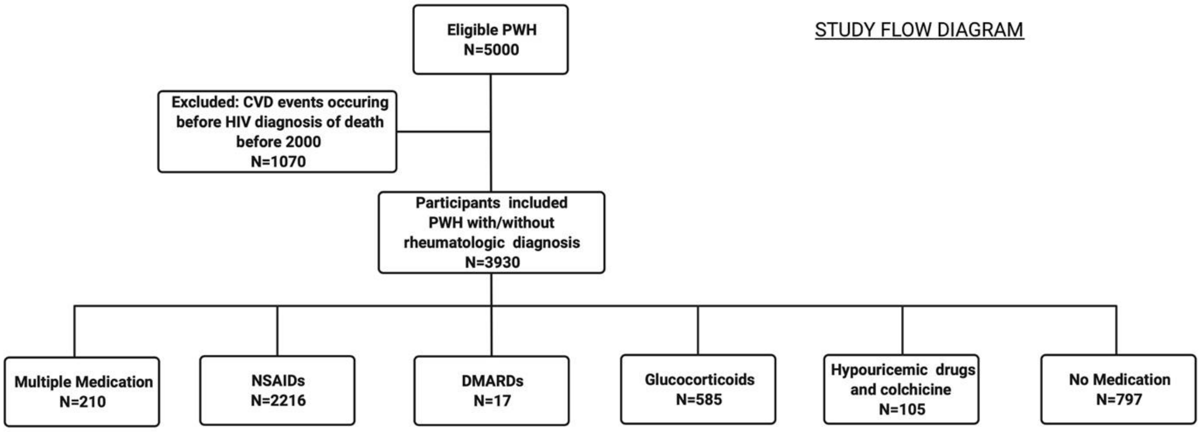

Figure S3. Legend: Study flow diagram showing breakdown of study participants excluded and those included in the analysis, classified by medication exposure category.
